# Harnessing the potentials of predictive microbiology in microbial food safety and quality research in Nigeria

**DOI:** 10.4155/fso.15.91

**Published:** 2016-01-15

**Authors:** Olumide A Odeyemi, Florence A Bamidele

**Affiliations:** 1 Ecology & Biodiversity Centre, Institute for Marine & Antarctic Studies, University of Tasmania, Australia; 2 Department of Biological sciences, School of Applied Science, Yaba College of Technology, Lagos, Nigeria

**Keywords:** foodborne diseases, mathematical modeling, microbial food safety, predictive microbiology

Food constitutes one of the most important routes of transmission of infectious diseases. Microbial safety and quality of food is a global health concern owing to the emergence and re-emergence of foodborne pathogens worldwide recently. Microbial pathogens are disease causing bacteria, viruses, fungi and protozoans [1,2].

Food-harboring microbial pathogens and microbial toxins can lead to foodborne disease [3]. Such unsafe foods, therefore, pose a global health threat and threaten economic loss, especially for those with compromised immune systems. Foodborne diseases are one of the 13 zoonoses responsible for over 2 billion human illnesses globally and result in 2.3 million deaths per year, mostly in developing countries [4]. Concerted efforts from governments, policy makers, scientists and general public in developing countries are required for both awareness and prevention of transmission of foodborne diseases.

## The global burden of foodborne illnesses

Governments of developing countries should provide funding and adequately equip healthcare systems. Scientific funding should be provided to investigate and understand the behavior, growth and survival of foodborne pathogens in the food matrix, and host–pathogen interactions. The outcome of such research will help in development of appropriate intervention strategies and rapid detection methods. Studies on burden and cost of foodborne diseases in each country should be carried out. Despite the effort and research funding of countries such as the USA, Canada, The Netherlands, Australia, New Zealand and France, such countries still have a high incidence of foodborne illnesses [5,6]. For example, in a study to estimate burden of foodborne illnesses in 2014, it was reported that one in eight Canadians become ill as a result of eating food contaminated with 30 different foodborne pathogens including Norovirus (1 million cases), *Clostridium perfringens* (1,770,000 cases), *Campylobacter* (1,450,000 cases) and nontyphoidal *Salmonella* (88,000 cases), while the CDC in the USA has reported that more than 47 million foodborne diseases, leading to over 1,250,000 hospitalizations and 3037 deaths, occur annually as a result of consuming contaminated food [5,6].

Developing countries, in particular, need to step up to the challenge of ensuring public health safety of their citizens [5]. Nigeria remains one of the most affected countries in the world due to poverty, poor sanitation, poor personal hygiene and low public awareness. The burden of foodborne diseases is global and, therefore, requires global effort in terms of collaboration, funding, awareness and commitment from various governments, especially developing nations and policy makers.

## Addressing food safety in Nigeria

Food safety awareness and education should be emphasized and encouraged among citizens globally. In particular, homes in developing countries contribute highly to foodborne disease outbreaks owing to contamination, lack of awareness, poor personal hygiene and improper food handling [7]. Training skills in state-of-the-art equipment used for rapid detection of foodborne pathogens and scientific collaboration with developed countries should also be encouraged.

Considering the huge economic burden of foodborne diseases, it is important that appropriate measures are taken to prevent foodborne disease outbreaks in Nigeria. The accurate statistical data for foodborne illnesses in Nigeria is still unknown, but has been reported to be endemic [8]. Preparedness, capacity building, intensive public awareness, creation of food safety databases, nationwide helplines and collaborative research efforts with developed countries are important measures in order to prevent foodborne disease outbreaks. Among the recent steps taken by the Nigerian government is inauguration of food safety committees comprising the Inter-Ministerial Committee on Food Safety (IMCFS) and the National Food Safety Management Committee (NFSMC), following a bill in 2014.

However, the more proactive steps highlighted above still need to be taken to ensure public health safety in Nigeria. There is a need to study and understand the behavior, survival and growth of foodborne pathogens in different food matrices using predictive modeling. Experts could be trained in this field using manpower resources available from countries such as the USA, the UK and Australia. Last, an autonomous national food safety research institute should also be established.

## Predictive microbiology & microbial food safety & quality research

Predictive microbiology has been used in developed countries such as the USA, Australia, Spain, Ireland, Iceland, Denmark, the UK and Belgium for shelf-life extension, food preservation, product development, improvement of industrial produce and understanding of behavior of various pathogens in the food matrix. For example, in Australia, predictive microbiology was used to increase the revenue of the red meat industry to AU$44 million over 30 years of research with an initial funding of AU$3.2 million provided by Meat and Livestock Australia (MLA) [9]. During the study, a tool known as Refrigeration Index was developed and validated for industrial use thereby lowering cost of compliance to regulations and training of >800 personnel in the meat industry.

Currently in developed countries, predictive microbiology is used for identification of hazard analysis and critical control points during production, and also for microbial risk assessment. Most of the models currently available are more suitable for use in developed countries. They cannot be used in countries like Nigeria, which is a seasonally damp and humid country. These climatic conditions provide suitable temperature for microbial growth in food, thereby causing food spoilage. In Nigeria, predictive microbiology can be used to study optimum temperature and environmental conditions suitable for microbial growth. Additionally, it can be used to optimize various microbial fermentation processes during production. It can be used to study and understand the survival and growth of bacteria and fungi in different food matrices.

Considering the huge economic risks associated with foodborne illnesses, the national food regulatory organization in Nigeria needs to use this technique to check compliance of food industries to safety guidelines as it can estimate change in microbial population and risk of consumers in consuming contaminated food. If well implemented, predictive microbiology can help food industry reduce cost of compliance to regulations. The potentials and benefits of predictive microbiology can be maximized through research funding, development of predictive microbiology software and database, capacity building and international scientific collaborations with scientists from developed countries.

## Conclusion & future perspective

Human survival depends on food. However, contaminated food and food products pose dangers to the health of consumers. There has been a global increase in foodborne disease outbreaks yearly, especially in developing countries such as Nigeria. Understanding microbial behavior in food matrices will help mitigate against foodborne disease outbreaks. Predictive microbiology as a field of food microbiology has been used in developed countries to understand microbial behavior in food, predict shelf life and to decide critical control points during food production processes. However, the potential of predictive microbiology is yet to be fully utilized in Nigeria. There is a need for capacity building in predictive microbiology and international scientific collaborations with experts from developed countries so as to harness the potentials of this technique.
